# Alcohol-Only and Drug/Medication-Only Product-Associated Facial Injury Encounters Across COVID-19 Periods: A Public CPSC NEISS Study, 2019–2023

**DOI:** 10.3390/cmtr19030035

**Published:** 2026-08-14

**Authors:** David M. Kachar, Christopher T. George, Andrea Ziegler, Eric Thorpe

**Affiliations:** 1 Chicago Medical School, Rosalind Franklin University of Medicine and Science, North Chicago, IL 60064, USA; christopher.george@my.rfums.org; 2 Department of Otolaryngology–Head & Neck Surgery, Loyola University Medical Center, Maywood, IL 60153, USA

**Keywords:** facial trauma, alcohol involvement, drug/medication involvement, COVID-19, NEISS, emergency department, consumer product-associated injury

## Abstract

Previous analyses of substance-involved craniofacial trauma have often combined alcohol and drug/medication involvement. We conducted a cross-sectional analysis comparing alcohol-only and drug/medication-only facial injury encounters in the public Consumer Product Safety Commission National Electronic Injury Surveillance System (NEISS), 2019–2023. Primary facial injuries (Body_Part = 76) were classified using the released Alcohol_Involved and Drug_Involved fields. Survey-weighted estimates and Taylor-linearized 95% confidence intervals incorporated the released PSU, stratum, and weight variables; design-based logistic regression evaluated code-4 hospitalization. Among 146,857 observed facial injury encounters, 4117 were alcohol-only and 2302 were drug/medication-only. Weighted annual alcohol-only proportions were stable (trend odds ratio [OR] per year, 0.99; 95% CI, 0.96–1.03; *p* = 0.716), whereas drug/medication-only proportions increased (OR, 1.15; 95% CI, 1.04–1.27; *p* = 0.009). Compared with alcohol-only encounters, drug/medication-only encounters involved older patients (weighted mean age, 63.1 vs. 47.6 years) and a lower weighted male proportion (55.6% vs. 67.5%). Weighted code-4 hospitalization was 23.1% versus 11.8%; the adjusted OR was 1.91 (95% CI, 1.45–2.53; *p* < 0.001). Adult-only and alternative-period sensitivity analyses supported the main conclusions. Separate classification of alcohol and drug/medication involvement revealed distinct demographic, temporal, and disposition patterns among NEISS-captured product-associated ED encounters.

## 1. Introduction

Facial trauma is a clinically relevant component of emergency department (ED) care and may result in functional, aesthetic, reconstructive, and psychosocial consequences extending beyond the acute injury [1,2]. Facial trauma may result in fractures, lacerations, and other soft-tissue injuries, some of which require procedural or surgical management. Some patients require hospital admission, operative intervention, or longitudinal follow-up, making accurate surveillance of facial injury patterns relevant to prevention and healthcare planning [3].

Alcohol and drug involvement have long been recognized as important contributors to facial injury incidence and severity [4,5]. Recent studies have addressed related but distinct populations. A NEISS analysis described alcohol-involved facial injuries [6], whereas a TriNetX study evaluated patients with facial fractures and temporally associated illicit-drug diagnoses across participating health care organizations [7]. The latter was not a NEISS analysis and did not use the exposure groups examined here. A separate NEISS study assessed controlled-substance involvement across burn, hand, and face injuries [8]. These studies support examining substance categories separately while avoiding direct equivalence across data sources, injury definitions, and exposure definitions.

Combining alcohol and drug/medication involvement into a single analytic group may obscure clinically meaningful differences. Alcohol-related facial trauma has typically been associated with younger, predominantly male patients and assault- or fall-related mechanisms, particularly in social settings [4,5]. The NEISS Drugs/Medication indicator may encompass prescription medications, illicit substances, or other drugs when their use contributed to the incident or injury severity, but the binary public-data field does not provide a standardized drug-class category [9]. Therefore, it is plausible that the two exposures differ in their typical age distribution, mechanism, severity, and admission risk. Without separating them, it is challenging to determine whether alcohol-involved and drug-involved facial injury encounters represent the same underlying patient population or distinct cohorts.

The COVID-19 pandemic provides a useful temporal context for evaluating whether observed encounter patterns changed during 2019–2023. Public health restrictions, health care utilization, and substance-use patterns changed over this interval [10,11,12,13,14,15]. Because the selected cut points are author-defined and the study is descriptive, the periods are treated as analytic time categories rather than causal pandemic exposures.

We therefore compared alcohol-only and drug/medication-only primary facial injury encounters captured in public CPSC NEISS from 2019 through 2023. The prespecified objectives were to estimate annual and period-specific proportions using the complete released survey design, compare demographic and injury characteristics, and estimate unadjusted and adjusted associations with NEISS disposition code 4. We also performed adult-only and alternative-period sensitivity analyses.

## 2. Materials and Methods

### 2.1. Study Design and Data Source

We conducted a cross-sectional analysis of the 2019–2023 public Consumer Product Safety Commission National Electronic Injury Surveillance System (CPSC NEISS) files [9,16]. NEISS is a stratified probability sample of U.S. hospital emergency departments and captures injuries meeting CPSC product-association and reporting criteria. The public files contained the primary sampling unit (PSU), stratum, and statistical weight for every included year; these variables were retained and used for all primary weighted estimates and variance calculations. This report follows the STROBE guidance for cross-sectional studies.

### 2.2. Case Selection

We screened all records dated 1 January 2019, through 31 December 2023, and selected encounters with primary Body_Part = 76 (face). This definition intentionally excludes records in which a facial injury was only a secondary diagnosis. No age restriction was used for the primary analysis. NEISS infant-age codes 201–223 were converted from months to years, and Age = 0 was treated as missing. An adult-only analysis restricted the two exposure groups to recoded age >=18 years.

### 2.3. Substance Involvement Definitions

Encounters were stratified into four mutually exclusive groups based on the NEISS Alcohol_Involved and Drug_Involved fields: alcohol-only (Alcohol_Involved = 1, Drug_Involved = 0), drug/medication-only (Drug_Involved = 1, Alcohol_Involved = 0), both alcohol and drug involvement (Alcohol_Involved = 1, Drug_Involved = 1), and neither (both = 0). According to the NEISS coding manual, Alcohol_Involved is coded when the record indicates that the patient consumed alcohol before or during the incident. Drugs/Medication is coded when the record indicates that use or regular use of a drug or medication contributed to the incident or the severity of the injury [9]. The binary public-data indicator does not provide a standardized drug-class variable. The term “drug/medication-only” is used throughout because it corresponds most closely to the scope of the NEISS field. The primary comparison was alcohol-only versus drug/medication-only encounters. The both and neither groups were retained for descriptive characterization but were not the focus of inferential testing.

### 2.4. COVID-19 Period Definitions

The primary analysis used three author-defined periods: pre-pandemic (January 2019–February 2020), early-pandemic (March 2020–June 2021), and later-pandemic/recovery (July 2021–December 2023). These labels are descriptive and do not assert that the pandemic caused observed differences. A sensitivity analysis used calendar-era categories of 2019, 2020–2021, and 2022–2023.

### 2.5. Variables and Outcomes

Patient characteristics included recoded age, sex, race, NEISS primary diagnosis, and injury location. The primary outcome was code-4 hospitalization, defined strictly as NEISS Disposition = 4; it should not be interpreted as all admissions or all severe outcomes. Diagnosis codes 57 (fracture) and 62 (internal organ injury) defined the severe-diagnosis covariate, although code 62 was absent after the primary-face restriction. An exploratory probable-fall indicator used case-insensitive narrative matches for FALL, FELL, SLIP, SLIPPED, TRIPP, or STUMBL. This keyword measure was not manually validated and was not treated as a definitive mechanism classification.

### 2.6. Statistical Analysis

Observed sample counts are unweighted. All primary proportions, means, totals, confidence intervals, hypothesis tests, and regression models incorporated the released NEISS PSU, stratum, and weight. We specified a Taylor-linearized survey design with PSU as the cluster, Stratum as the stratum, Weight as the sampling weight, nesting enabled, and isolated-PSU adjustment; analytic cohorts were evaluated as domains of the complete five-year design. Cumulative encounter estimates used the released, unscaled annual weights. Rao-Scott tests compared categorical distributions, and design-based contrasts compared means and proportions. Survey-weighted logistic regression estimated odds ratios (ORs) and 95% CIs. The main code-4 hospitalization model compared drug/medication-only with alcohol-only encounters and adjusted for continuous age, sex, primary period, and severe diagnosis. Period-specific models omitted the period covariate. Annual trend models used a continuous calendar year. Complete-case model counts, rank, convergence, event counts, leverage, and standardized condition diagnostics were checked. Two-sided *p* < 0.05 was considered statistically significant.

Data import, recoding, audit outputs, and figure generation were performed in Python 3.14.2 using pandas 2.3.3 and related packages [17]. Survey analyses were performed in R 4.6.0 using survey 4.5 [18,19]. The complete version-pinned code and run instructions are provided in Supplementary File S1.

Generative artificial intelligence (OpenAI Codex, exact model/configuration as recorded in project logs; accessed May–June 2026) was used to assist with drafting portions of the Python analysis code and figure-generation code. It was also used to assist with manuscript wording. The authors independently reviewed the code, executed the complete analysis, compared the generated outputs with the underlying data, edited all resulting text and figures, and take responsibility for the final analysis and manuscript.

## 3. Results

### 3.1. Study Cohort

The five annual files contained 1,670,132 observed NEISS records, of which 146,857 met the primary-face criterion. The mutually exclusive groups comprised 4117 alcohol-only, 2302 drug/medication-only, 330 both, and 140,108 neither encounters. Primary comparisons focused on the alcohol-only and drug/medication-only groups (Table 1).

### 3.2. Temporal Trends in Substance-Involved Facial Injury Encounters

**Figure 1:**
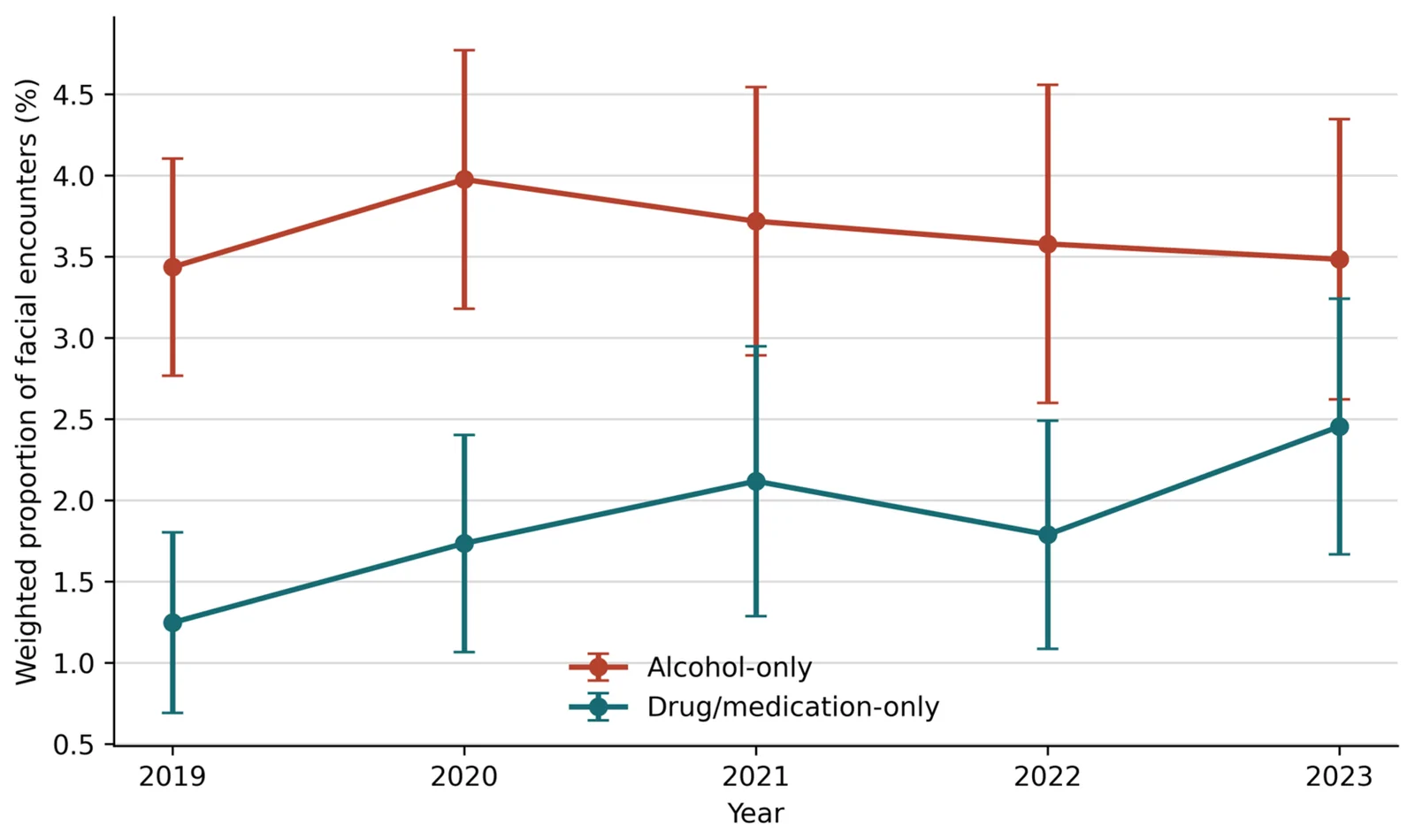
Survey-weighted annual proportions of alcohol-only and drug/medication-only encounters among NEISS-captured primary facial injury encounters, 2019–2023. Error bars show Taylor-linearized 95% confidence intervals. The figure does not imply causal effects of pandemic conditions.

Weighted alcohol-only proportions were 3.44%, 3.98%, 3.72%, 3.58%, and 3.48% from 2019 through 2023; the design-based linear trend was not significant (OR per year, 0.99; 95% CI, 0.96–1.03; *p* = 0.716). Weighted drug/medication-only proportions were 1.25%, 1.73%, 2.12%, 1.79%, and 2.45%, respectively, with evidence of an increasing trend (OR per year, 1.15; 95% CI, 1.04–1.27; *p* = 0.009) (Table 2; Figure 1). These are proportions of NEISS-captured primary facial injury encounters, not population incidence rates.

### 3.3. Demographic Differences Between Substance Groups

**Figure 2:**
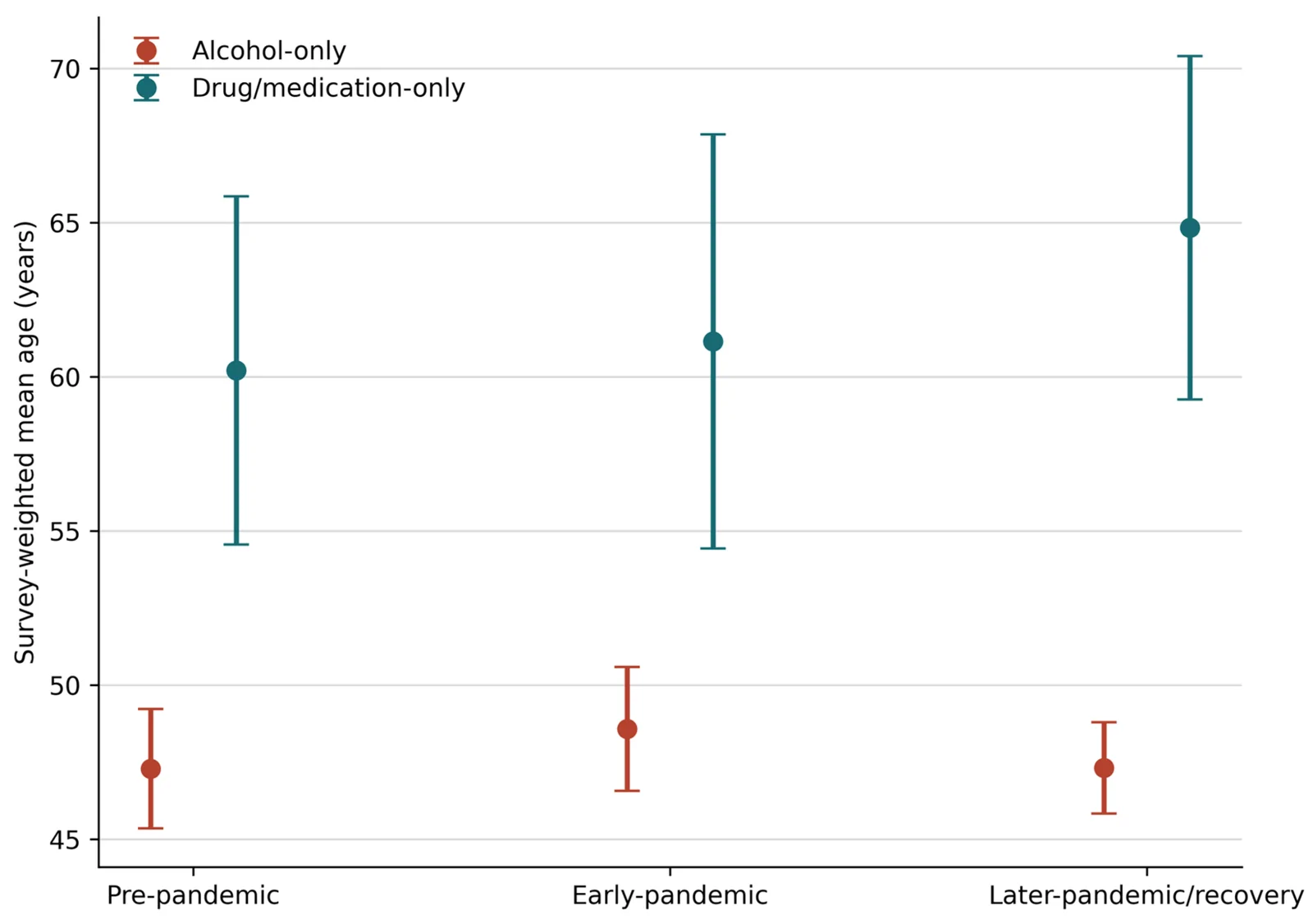
Survey-weighted mean age for alcohol-only and drug/medication-only NEISS primary facial injury encounters across author-defined periods, 2019–2023. Error bars show Taylor-linearized 95% confidence intervals. Overall mean difference: 15.5 years (95% CI, 10.1–20.8; Table 1).

Drug/medication-only encounters involved older patients than alcohol-only encounters (weighted mean age, 63.1 years [95% CI, 57.6–68.7] vs. 47.6 years [95% CI, 46.2–49.0]; difference, 15.5 years [95% CI, 10.1–20.8]; *p* < 0.001). The weighted male proportion was lower in the drug/medication-only group (55.6% [95% CI, 50.6–60.5]) than in the alcohol-only group (67.5% [95% CI, 64.7–70.3]); risk difference, −11.9 percentage points (95% CI, −16.2 to −7.7; *p* < 0.001). Period-specific weighted ages are shown in Figure 2.

### 3.4. Diagnosis and Mechanism Patterns

The survey-weighted distribution of selected primary diagnoses differed between groups (Rao-Scott *p* < 0.001). Alcohol-only encounters had a higher weighted fracture proportion than drug/medication-only encounters (21.0% vs. 12.0%) and a higher laceration proportion (46.4% vs. 34.6%). Drug/medication-only encounters had higher contusion/abrasion (31.9% vs. 23.5%) and hematoma (11.4% vs. 3.9%) proportions. Race and injury-location distributions also differed in design-based global tests (*p* = 0.022 and *p* < 0.001, respectively) (Table 1).

The exploratory keyword-defined probable-fall proportion was 81.8% (95% CI, 79.5–84.2) for alcohol-only and 78.5% (95% CI, 71.9–85.1) for drug/medication-only encounters. The difference was not statistically significant (risk difference, −3.3 percentage points; 95% CI, −9.2 to 2.6; *p* = 0.269). Because the algorithm was not manually validated, this result is descriptive and exploratory.

### 3.5. Code-4 Hospitalization

Weighted code-4 hospitalization was higher for drug/medication-only than alcohol-only encounters in each primary period: 27.2% versus 11.6% pre-pandemic, 23.7% versus 13.7% early-pandemic, and 21.6% versus 11.0% later-pandemic/recovery. Adjusted period-specific ORs were 2.51 (95% CI, 1.62–3.89), 1.83 (95% CI, 1.10–3.03), and 1.76 (95% CI, 1.43–2.16), respectively (Table 3).

Overall weighted code-4 hospitalization was 23.1% (95% CI, 19.2–27.0) in drug/medication-only encounters and 11.8% (95% CI, 10.0–13.7) in alcohol-only encounters. The unadjusted survey OR was 2.23 (95% CI, 1.69–2.94). After adjustment for age, sex, primary period, and severe diagnosis, the OR was 1.91 (95% CI, 1.45–2.53; *p* < 0.001). The model used 6415 complete cases among 6419 two-group encounters; four records had missing recoded age. Convergence, full rank, event-count, leverage, and condition diagnostics were acceptable.

### 3.6. Sensitivity Analyses

The adult-only analysis included 4094 alcohol-only and 2197 drug/medication-only observed encounters. The adjusted survey OR for code-4 hospitalization was 1.96 (95% CI, 1.47–2.60; *p* < 0.001), and annual trends remained nonsignificant for alcohol-only (OR per year, 0.99; 95% CI, 0.95–1.02; *p* = 0.433) and positive for drug/medication-only encounters (OR, 1.14; 95% CI, 1.03–1.27; *p* = 0.012). Under alternative calendar eras, adjusted hospitalization ORs were 2.56 (95% CI, 1.53–4.31) for 2019, 2.06 (95% CI, 1.40–3.05) for 2020–2021, and 1.56 (95% CI, 1.19–2.04) for 2022–2023. Both analyses supported the main conclusions (Supplementary File S3).

## 4. Discussion

Using the complete released NEISS survey design, this study found distinct patterns for alcohol-only and drug/medication-only primary facial injury encounters. Alcohol-only weighted proportions showed no significant annual trend, whereas drug/medication-only proportions increased over 2019–2023. Patients in the drug/medication-only group were older on average, less often male, and more likely to have code-4 hospitalization after covariate adjustment.

The findings reinforce the methodological value of keeping alcohol and drug/medication indicators separate. The NEISS alcohol-focused study [6], the TriNetX illicit-drug facial-fracture study [7], and the broader NEISS controlled-substance study [8] address different case definitions and data-generating systems. Reference [7] examined a matched healthcare network cohort with facial-fracture and illicit-drug diagnoses, whereas the present study examined NEISS primary facial injuries classified by released involvement flags. Therefore, direct numerical comparison is inappropriate.

The weighted age difference was large and persisted in the adult-only analysis. Diagnosis patterns also differed: fractures and lacerations were more common in alcohol-only encounters, whereas contusions and hematomas were more common in drug/medication-only encounters. NEISS does not identify specific drugs within the Drug_Involved field, and this study cannot attribute hospitalization to intoxication, withdrawal, comorbidity, injury complexity, or any other clinical pathway.

These results are surveillance findings rather than clinical treatment recommendations. Separate recording and analysis of alcohol and drug/medication involvement may preserve differences that are lost in a combined substance-involvement category. The exploratory probable-fall comparison was not statistically significant and should not be used as a validated mechanism estimate.

The persistence of higher drug/medication-only proportions through 2023 warrants continued surveillance, but the temporal associations do not establish pandemic causation. The alternative calendar-era analysis produced the same qualitative hospitalization conclusion, reducing dependence on the selected primary cut points.

### Limitations

This study has several limitations. Public CPSC NEISS represents product-associated injuries treated in sampled U.S. emergency departments and meeting NEISS reporting criteria; it does not capture all facial trauma, all ED visits, inpatient-only care, or non-product-associated events. Estimates should therefore be described as national estimates of NEISS-captured product-associated ED encounters, not all U.S. facial injuries. Alcohol_Involved and Drug_Involved are broad surveillance flags subject to documentation and coding error. Drug_Involved does not identify the substance, dose, prescription status, indication, or whether a medication contributed causally. The mutually exclusive groups do not measure use disorder, intoxication, or withdrawal. Restriction to primary Body_Part = 76 excludes secondary facial injuries. Disposition code 4 is a specific NEISS code and is not equivalent to every form of admission or severity. Residual confounding remains possible, including comorbidity and injury circumstances unavailable in public NEISS. The probable-fall keyword algorithm was not manually validated. Cumulative totals use unscaled annual weights and should not be interpreted as unique persons. Finally, author-defined periods support temporal description but not causal attribution to COVID-19.

## 5. Conclusions

Among NEISS-captured product-associated primary facial injury encounters from 2019 through 2023, alcohol-only weighted proportions were stable while drug/medication-only proportions increased. Drug/medication-only encounters involved older patients and had higher adjusted odds of code-4 hospitalization than alcohol-only encounters. Adult-only and alternative-period sensitivity analyses supported these conclusions. The findings favor separate surveillance classification of alcohol and drug/medication involvement while remaining subject to the scope and coding limitations of public NEISS.

## Figures and Tables

**Table 1:** Characteristics of NEISS facial injury encounters by substance involvement group, 2019–2023.

Characteristic	Alcohol-Only	Drug/Medication-Only	Effect/Contrast (95% CI)	Survey-Adjusted *p*
Observed sample, n	4117	2302		
Weighted encounter estimate (95% CI)	177,573 (136,208 to 218,939)	91,113 (60,844 to 121,382)		
Mean age, years	47.6 (46.2 to 49.0)	63.1 (57.6 to 68.7)	15.5 (10.1 to 20.8) years	<0.001
Male, %	67.5 (64.7 to 70.3)	55.6 (50.6 to 60.5)	−11.9 (−16.2 to −7.7) percentage points	<0.001
Code-4 hospitalization, %	11.8 (10.0 to 13.7)	23.1 (19.2 to 27.0)	11.3 (7.0 to 15.5) percentage points	<0.001
Exploratory keyword-defined probable fall, %	81.8 (79.5 to 84.2)	78.5 (71.9 to 85.1)	−3.3 (−9.2 to 2.6) percentage points	0.269
Laceration, %	46.4 (43.8 to 48.9)	34.6 (31.3 to 38.0)		<0.001
Contusion/abrasion, %	23.5 (21.5 to 25.5)	31.9 (29.0 to 34.9)		
Fracture, %	21.0 (19.3 to 22.8)	12.0 (10.1 to 14.0)		
Hematoma, %	3.9 (3.1 to 4.7)	11.4 (9.4 to 13.4)		
White, %	56.2 (45.7 to 66.6)	67.6 (57.4 to 77.9)		0.022
Black/African American, %	16.3 (6.2 to 26.4)	14.1 (5.7 to 22.5)		
Not specified, %	23.6 (12.1 to 35.0)	15.4 (7.0 to 23.8)		
Home, %	40.0 (32.3 to 47.8)	52.8 (43.9 to 61.8)		<0.001
Street/highway, %	9.3 (6.3 to 12.3)	3.2 (0.9 to 5.5)		
Other public property, %	15.9 (13.6 to 18.1)	20.2 (17.1 to 23.2)		

Values are observed counts or survey-weighted estimates with Taylor-linearized 95% confidence intervals. Diagnosis, race, and location *p* values are survey-adjusted omnibus/global Rao-Scott tests and appear once per distribution; individual rows within those multi-category distributions do not represent separate omnibus tests. Age, sex, code-4 hospitalization, and exploratory probable-fall *p* values are their corresponding survey-adjusted group comparisons. The probable-fall measure is an exploratory, unvalidated narrative-keyword definition. Categories may not total 100% because only selected levels are displayed and because of rounding.

**Table 2:** Annual proportion of alcohol-only and drug/medication-only NEISS facial injury encounters, 2019–2023.

Year	Alcohol-Only Observed n	Alcohol-Only Weighted Estimate (95% CI)	Alcohol-Only Weighted Proportion, % (95% CI)	Drug/Medication-Only Observed n	Drug/Medication-Only Weighted Estimate (95% CI)	Drug/Medication-Only Weighted Proportion, % (95% CI)
2019	842	36,766 (28,628 to 44,904)	3.44 (2.77 to 4.11)	340	13,338 (7799 to 18,876)	1.25 (0.69 to 1.80)
2020	827	34,821 (26,750 to 42,892)	3.98 (3.18 to 4.77)	385	15,192 (9470 to 20,913)	1.73 (1.07 to 2.40)
2021	823	34,365 (26,134 to 42,596)	3.72 (2.89 to 4.54)	500	19,571 (12,027 to 27,115)	2.12 (1.29 to 2.95)
2022	784	36,329 (25,843 to 46,816)	3.58 (2.60 to 4.56)	449	18,153 (10,488 to 25,818)	1.79 (1.09 to 2.49)
2023	841	35,292 (25,829 to 44,754)	3.48 (2.62 to 4.35)	628	24,860 (16,125 to 33,595)	2.45 (1.67 to 3.24)
Trend OR per year (95% CI; *p*)			0.99 (0.96 to 1.03); 0.716			1.15 (1.04 to 1.27); 0.009

Observed n values are unweighted. Encounter estimates and proportions use the full released NEISS survey design and are presented with Taylor-linearized 95% confidence intervals. Trend ORs are from design-based logistic models with the calendar year entered continuously.

**Table 3:** Unadjusted and adjusted odds of code-4 hospitalization for drug/medication-only versus alcohol-only NEISS facial injury encounters.

Period	Alcohol-Only Code-4 Hospitalization, % (95% CI)	Drug/Medication-Only Code-4 Hospitalization, % (95% CI)	Unadjusted OR (95% CI)	Adjusted OR (95% CI)	Adjusted *p*	Model
Pre-pandemic	11.6 (8.6 to 14.6)	27.2 (22.6 to 31.9)	2.86 (2.02 to 4.06)	2.51 (1.62 to 3.89)	<0.001	Full NEISS design: PSU, stratum, and weight
Early-pandemic	13.7 (10.4 to 17.0)	23.7 (18.0 to 29.5)	1.94 (1.20 to 3.12)	1.83 (1.10 to 3.03)	0.019	Full NEISS design: PSU, stratum, and weight
Later-pandemic/recovery	11.0 (8.8 to 13.1)	21.6 (17.3 to 25.8)	2.23 (1.75 to 2.85)	1.76 (1.43 to 2.16)	<0.001	Full NEISS design: PSU, stratum, and weight
Overall	11.8 (10.0 to 13.7)	23.1 (19.2 to 27.0)	2.23 (1.69 to 2.94)	1.91 (1.45 to 2.53)	<0.001	Full NEISS design: PSU, stratum, and weight

Percentages and ORs use the full released NEISS survey design. Adjusted models include continuous age, sex, and severe diagnosis; the overall model additionally includes primary period. Code-4 hospitalization means NEISS Disposition = 4. CI—confidence interval; OR—odds ratio; PSU—primary sampling unit.

## Data Availability

The annual public CPSC NEISS datasets analyzed in this study are publicly available through the U.S. Consumer Product Safety Commission [16]. The complete revised analysis code and run instructions are provided as Supplementary File S1. Derived sensitivity-analysis results are provided as Supplementary File S3.

## Supplementary Materials

The following supporting information can be downloaded at: https://www.mdpi.com/article/10.3390/cmtr19030035/s1, Supplementary File S1 contains the complete Python and R analysis code, version information, and run instructions; Supplementary File S2 contains the completed STROBE cross-sectional checklist; Supplementary File S3 contains adult-only and alternative calendar-era sensitivity-analysis results.
