# Supplementary material for: Alcohol-Only and Drug/Medication-Only Product-Associated Facial Injury Encounters Across COVID-19 Periods: A Public CPSC NEISS Study, 2019–2023
Source: Craniomaxillofac Trauma Reconstr. 2026 Aug 14;19(3):35. doi: 10.3390/cmtr19030035 (PMC13510705; doi:10.3390/cmtr19030035)
Supplement: Supplementary file 1 [file cmtr-19-00035-s001.zip › Supplementary_File_S2_STROBE_Checklist.pdf]

STROBE Cross-Sectional Checklist

Completed for CMTR manuscript cmtr-4434359

**Manuscript**	cmtr-4434359
**Study design**	Cross-sectional analysis of ED encounters
**Final manuscript**	10 pages; line numbers 1-350

Checklist source: official STROBE checklist for cross-sectional studies. Locations refer to the final clean revised manuscript PDF generated on 31 July 2026.

Item	Recommendation	Reported	Page/line location	How addressed
1(a)	Indicate the study's design with a commonly used term in the title or abstract.	Yes	p. 1, line 13	The abstract identifies the design as a cross-sectional analysis.
1(b)	Provide an informative and balanced summary of what was done and what was found.	Yes	p. 1, lines 11-30	Structured narrative abstract reports objective, methods, principal estimates with CIs, sensitivity conclusion, and scope.
2	Explain the scientific background and rationale.	Yes	pp. 1-2, lines 34-66	Background explains facial-trauma relevance, prior substance research, exposure separation, and temporal context.
3	State specific objectives, including any prespecified hypotheses.	Yes	p. 2, lines 67-72	Objectives cover weighted temporal estimates, group comparisons, hospitalization, and sensitivity analyses.
4	Present key elements of study design early in the paper.	Yes	p. 2, lines 73-82	Cross-sectional public NEISS design and released survey variables are stated at the start of Methods.
5	Describe setting, locations, and relevant dates.	Yes	p. 2, lines 75-82; p. 3, lines 104-109	U.S. sampled ED setting, 2019-2023 dates, and primary/alternative periods are reported.
6(a)	Give eligibility criteria and sources and methods of participant selection.	Yes	pp. 2-3, lines 83-103	Primary-face selection, dates, age handling, and mutually exclusive exposure groups are defined.
6(b)	For matched studies, give matching criteria and numbers.	N/A	Not applicable	This is not a matched study.

Item	Recommendation	Reported	Page/line location	How addressed
7	Clearly define outcomes, exposures, predictors, confounders, and effect modifiers.	Yes	p. 3, lines 90-118 and 127-130	Exposure groups, code-4 outcome, age, sex, period, diagnosis, and severe-diagnosis covariate are defined.
8	Give sources of data and details of assessment methods.	Yes	pp. 2-4, lines 75-137	Public CPSC NEISS source, released coding fields, recoding, exploratory fall keywords, and survey analysis are described.
9	Describe efforts to address potential sources of bias.	Yes	p. 3, lines 119-133; p. 7, lines 219-227; p. 8, lines 257-273	Full released survey design, covariate adjustment, sensitivity analyses, and limitations address major bias sources.
10	Explain how the study size was arrived at.	Yes	pp. 2-3, lines 84-89; p. 4, lines 146-149	All eligible records in the five complete annual files were screened; no sampling-based target size was selected.
11	Explain how quantitative variables were handled.	Yes	pp. 2-3, lines 84-89; p. 3, lines 104-109 and 127-131	Corrected age is continuous in models, infant age is recoded, and period and adult thresholds are defined.
12(a)	Describe all statistical methods, including control for confounding.	Yes	p. 3, lines 119-133	Survey design, tests, contrasts, logistic models, covariates, annual trends, diagnostics, and significance threshold are reported.
12(b)	Describe methods used to examine subgroups and interactions.	Yes	pp. 2-3, lines 84-89; p. 3, lines 104-109 and 129-131; p. 7, lines 219-227	Period-specific models, adult-only analysis, and alternative calendar eras are described and reported.
12(c)	Explain how missing data were addressed.	Yes	pp. 2-3, lines 84-89; p. 3, lines 131-133; p. 7, lines 212-218	Age = 0 is defined as missing; complete-case diagnostics were checked; the main model used 6,415 of 6,419 encounters and excluded four records with missing recoded age.
12(d)	If applicable, describe analytical methods taking account of sampling strategy.	Yes	p. 3, lines 119-127	PSU clustering, stratification, weights, nesting, isolated-PSU handling, domain analysis, and unscaled totals are specified.

Item	Recommendation	Reported	Page/line location	How addressed
12(e)	Describe sensitivity analyses.	Yes	pp. 2-3, lines 84-89; p. 3, lines 104-109; p. 7, lines 219-227	Adult-only and alternative calendar-era analyses are prespecified and reported; full results are in Supplementary File S3.
13(a)	Report numbers at each stage of the study.	Yes	p. 4, lines 146-149; p. 7, lines 212-227	Raw screened count, facial cohort, exposure groups, model complete cases, and adult counts are reported.
13(b)	Give reasons for non-participation at each stage.	Yes	pp. 2-3, lines 84-89; p. 7, lines 215-217	The eligibility restriction and the model exclusion of four records with missing recoded age are stated; participation is not applicable to public records.
13(c)	Consider use of a flow diagram.	No	Not used; counts in text	A flow diagram was not necessary because selection consists of one primary-body-part restriction and transparent group classification.
14(a)	Give characteristics of study participants and information on exposures and confounders.	Yes	Table 1, p. 4	Observed n, weighted totals, age, sex, diagnosis, race, location, fall measure, and hospitalization are presented with CIs.
14(b)	Indicate number of participants with missing data for each variable of interest.	Yes	p. 7, lines 212-218	The main model used 6,415 of 6,419 encounters; four records had missing recoded age, and no other model-variable exclusions occurred.
14(c)	Summarize follow-up time.	N/A	Not applicable	Cross-sectional ED encounter analysis has no longitudinal follow-up.
15	Report numbers of outcome events or summary measures.	Yes	Tables 1-3, pp. 4-7	Weighted proportions, means, totals, and code-4 hospitalization estimates are presented with 95% CIs.
16(a)	Give unadjusted and adjusted estimates with precision and clarify confounders.	Yes	Table 3, pp. 6-7; p. 7, lines 208-218	Unadjusted and adjusted ORs with 95% CIs and model covariates are reported.
16(b)	Report category boundaries when continuous variables were categorized.	Yes	pp. 2-3, lines 84-89; p. 3, lines 104-109	Adult age >=18 and all period boundaries are explicit.

Item	Recommendation	Reported	Page/line location	How addressed
16(c)	Consider translating relative risk into absolute risk for a meaningful time period.	Yes	Table 3, pp. 6-7	Absolute weighted code-4 hospitalization proportions accompany ORs for each group and period.
17	Report other analyses, including subgroup and sensitivity analyses.	Yes	p. 7, lines 219-227; Supplementary File S3	Adult-only and alternative calendar-era estimates are summarized and supplied in full.
18	Summarize key results with reference to study objectives.	Yes	pp. 7-8, lines 229-256	Discussion summarizes temporal, demographic, diagnosis, and hospitalization findings.
19	Discuss limitations, including direction and magnitude of potential bias.	Yes	p. 8, lines 257-273	Scope, coding, exposure, outcome, confounding, fall validation, weights, and causal limits are discussed.
20	Give a cautious overall interpretation considering objectives, limitations, multiplicity, and other evidence.	Yes	pp. 7-8, lines 229-281	Interpretation is explicitly surveillance-focused, noncausal, and bounded by NEISS and sensitivity findings.
21	Discuss generalisability of the study results.	Yes	p. 8, lines 257-281	The manuscript limits inference to NEISS-captured product-associated ED encounters and avoids all-injury claims.
22	Give the source of funding and role of funders.	Yes	p. 8, line 291	The Funding statement reports that no external funding was received.
