# Supplementary material for: Alcohol-Only and Drug/Medication-Only Product-Associated Facial Injury Encounters Across COVID-19 Periods: A Public CPSC NEISS Study, 2019–2023
Source: Craniomaxillofac Trauma Reconstr. 2026 Aug 14;19(3):35. doi: 10.3390/cmtr19030035 (PMC13510705; doi:10.3390/cmtr19030035)
Supplement: Supplementary file 1 [file cmtr-19-00035-s001.zip › Supplementary_File_S3.pdf]

Section	Metric	Group	Estimate	CI low	CI high	P value
Cohort	Observed n	Alcohol-only	4,094			
Characteristics	Weighted encounter estimate	Alcohol-only	176,496.9	135,306.7	217,687.1	
Characteristics	Mean age	Alcohol-only	47.80	46.37	49.23	
Characteristics	Male	Alcohol-only	67.61%	64.75%	70.46%	
Characteristics	Code-4 hospitalization	Alcohol-only	11.85%	10.00%	13.70%	
Annual proportions	2019 proportion	Alcohol-only	6.74%	5.66%	7.81%	
Annual proportions	2020 proportion	Alcohol-only	7.45%	6.27%	8.64%	
Annual proportions	2021 proportion	Alcohol-only	6.97%	5.88%	8.06%	
Annual proportions	2022 proportion	Alcohol-only	6.79%	5.37%	8.20%	
Annual proportions	2023 proportion	Alcohol-only	6.61%	5.39%	7.82%	
Cohort	Observed n	Drug/medication-only	2,197			
Characteristics	Weighted encounter estimate	Drug/medication-only	88,272.9	58,285.2	118,260.6	
Characteristics	Mean age	Drug/medication-only	64.73	59.35	70.11	
Characteristics	Male	Drug/medication-only	55.72%	50.52%	60.92%	
Characteristics	Code-4 hospitalization	Drug/medication-only	23.64%	19.59%	27.70%	
Annual proportions	2019 proportion	Drug/medication-only	2.40%	1.42%	3.37%	
Annual proportions	2020 proportion	Drug/medication-only	3.16%	2.00%	4.32%	
Annual proportions	2021 proportion	Drug/medication-only	3.86%	2.42%	5.29%	
Annual proportions	2022 proportion	Drug/medication-only	3.31%	2.04%	4.57%	
Annual proportions	2023 proportion	Drug/medication-only	4.53%	3.15%	5.91%	

Hospitalization model	Full design unadjusted	Drug/medication-only vs Alcohol-only	2.30	1.74	3.04	<0.001
Hospitalization model	Full design adjusted	Drug/medication-only vs Alcohol-only	1.96	1.47	2.60	<0.001
Annual trend	Per one-year increase	Alcohol-only	0.99	0.95	1.02	0.433
Annual trend	Per one-year increase	Drug/medication-only	1.14	1.03	1.27	0.012

Period	Group	Metric	Estimate	CI low	CI high	P value	Method
2019	Alcohol-only	Proportion of facial encounters	3.44%	2.77%	4.11%		Full NEISS design
2019	Alcohol-only	Code-4 hospitalization	10.90%	7.94%	13.85%		Full NEISS design
2019	Drug/medication-only	Proportion of facial encounters	1.25%	0.69%	1.80%		Full NEISS design
2019	Drug/medication-only	Code-4 hospitalization	25.49%	19.13%	31.85%		Full NEISS design
2019	Drug/medication-only vs Alcohol-only	Full design unadjusted period-specific	2.80	1.85	4.24	<0.001	Full NEISS design
2019	Drug/medication-only vs Alcohol-only	Full design adjusted period-specific	2.56	1.53	4.31	<0.001	Full NEISS design
2020-2021	Alcohol-only	Proportion of facial encounters	3.84%	3.07%	4.62%		Full NEISS design
2020-2021	Alcohol-only	Code-4 hospitalization	12.81%	10.21%	15.42%		Full NEISS design
2020-2021	Drug/medication-only	Proportion of facial encounters	1.93%	1.20%	2.67%		Full NEISS design
2020-2021	Drug/medication-only	Code-4 hospitalization	26.35%	20.23%	32.47%		Full NEISS design
2020-2021	Drug/medication-only vs Alcohol-only	Full design unadjusted period-specific	2.41	1.62	3.59	<0.001	Full NEISS design
2020-2021	Drug/medication-only vs Alcohol-only	Full design adjusted period-specific	2.06	1.40	3.05	<0.001	Full NEISS design
2022-2023	Alcohol-only	Proportion of facial encounters	3.53%	2.62%	4.44%		Full NEISS design
2022-2023	Alcohol-only	Code-4 hospitalization	11.34%	8.68%	14.01%		Full NEISS design
2022-2023	Drug/medication-only	Proportion of facial encounters	2.12%	1.40%	2.84%		Full NEISS design
2022-2023	Drug/medication-only	Code-4 hospitalization	19.70%	15.84%	23.56%		Full NEISS design
2022-2023	Drug/medication-only vs Alcohol-only	Full design unadjusted period-specific	1.92	1.46	2.52	<0.001	Full NEISS design
2022-2023	Drug/medication-only vs Alcohol-only	Full design adjusted period-specific	1.56	1.19	2.04	0.001	Full NEISS design
